# Designing a Chatbot for a Brief Motivational Interview on Stress Management: Qualitative Case Study

**DOI:** 10.2196/12231

**Published:** 2019-04-16

**Authors:** SoHyun Park, Jeewon Choi, Sungwoo Lee, Changhoon Oh, Changdai Kim, Soohyun La, Joonhwan Lee, Bongwon Suh

**Affiliations:** 1 Human Centered Computing Lab. Seoul National University Seoul Republic of Korea; 2 Human Computer Interaction + Design Lab. Seoul National University Seoul Republic of Korea; 3 Department of Education Seoul National University Seoul Republic of Korea; 4 Center for Campus Life and Culture Seoul National University Seoul Republic of Korea

**Keywords:** motivational interviewing, mental health, conversational agents, stress management

## Abstract

**Background:**

In addition to addiction and substance abuse, motivational interviewing (MI) is increasingly being integrated in treating other clinical issues such as mental health problems. Most of the many technological adaptations of MI, however, have focused on delivering the action-oriented treatment, leaving its relational component unexplored or vaguely described. This study intended to design a conversational sequence that considers both technical and relational components of MI for a mental health concern.

**Objective:**

This case study aimed to design a conversational sequence for a brief motivational interview to be delivered by a Web-based text messaging application (*chatbot*) and to investigate its conversational experience with graduate students in their coping with stress.

**Methods:**

A brief conversational sequence was designed with varied combinations of MI skills to follow the 4 processes of MI. A Web-based text messaging application, Bonobot, was built as a research prototype to deliver the sequence in a conversation. A total of 30 full-time graduate students who self-reported stress with regard to their school life were recruited for a survey of demographic information and perceived stress and a semistructured interview. Interviews were transcribed verbatim and analyzed by Braun and Clarke’s thematic method. The themes that reflect the process of, impact of, and needs for the conversational experience are reported.

**Results:**

Participants had a high level of perceived stress (mean 22.5 [SD 5.0]). Our findings included the following themes: Evocative Questions and Clichéd Feedback; Self-Reflection and Potential Consolation; and Need for Information and Contextualized Feedback. Participants particularly favored the relay of evocative questions but were less satisfied with the agent-generated reflective and affirming feedback that filled in-between. Discussing the idea of change was a good means of reflecting on themselves, and some of Bonobot’s encouragements related to graduate school life were appreciated. Participants suggested the conversation provide informational support, as well as more contextualized feedback.

**Conclusions:**

A conversational sequence for a brief motivational interview was presented in this case study. Participant feedback suggests sequencing questions and MI-adherent statements can facilitate a conversation for stress management, which may encourage a chance of self-reflection. More diversified sequences, along with more contextualized feedback, should follow to offer a better conversational experience and to confirm any empirical effect.

## Introduction

### Background

In recent years, there has been a soaring number of technological adaptations of motivational interviewing (MI) [1]. Most of them, however, focus on changing problematic physical health and lifestyle behaviors (eg, [2-14]). This may be due to the fact that MI primarily targets behavior change and was originally introduced to treat substance abuse, such as addiction and drinking problems [15]. However, recent studies include MI in mental health issues, such as anxiety, depression, and other related problems (eg, [16-22]). It is increasingly acknowledged that MI can be used in a broader and more flexible context concerning ambivalence in change [16]. Most recently, MI took the means of life coaching for college students to cope with stress, yielding positive client experiences in stress reduction [23].

Whether MI is used in treating physical or mental health problems, it is stressed that the counsellor maintain a good MI spirit, that is, the counsellor being relationally adept in expressing empathy and responding to client resistance, in addition to the technical approach [24,25]. However, most technological adaptations of MI have limited descriptions of how the relational component of MI was resolved in their interventions [1]. If MI were to be used as an instrument for psychotherapy, its relational component should be translated in a proper manner to maneuver the treatment to a successful outcome [25]. Though recent studies have attempted to autogenerate words of empathy for a counseling conversation (eg, [26,27]), they provide insufficient description as to how the relational aspect of the conversation should be strategically managed, with much less regard to the specific relational skills.

To address the above problem, this study aimed to incorporate both technical and relational components of MI in a conversational sequence for a brief motivational interview and to investigate its conversational experience on a Web-based text messaging application (*chatbot*). Applying the summons-answer sequence [28], we have built a chatbot that delivers an ordered sequence of MI skills to follow the 4 processes of MI [29] in a conversation with a human user. We focused on the communication between the chatbot and the user, where a smooth interaction is required. The recent mobile chatbot apps that provide therapy (eg, [30-32]) mostly focus on identifying symptoms and providing treatment, leaving the communicative process less attended. Our study has contributed a potential technique and the user conversational experience of translating MI components in a computational manner to broadly inform future Web-based and mobile gadgets that may involve conversational encounters for mental health care.

In this study, a Web-based text messaging application that delivers a brief motivational interview for stress reduction was presented to a group of graduate students for a case study. Graduate students are reported with serious risks of a mental health crisis [33], and a large portion of the graduate student population already ails with mental illnesses such as anxiety and depression. Most of them are reported to have low life satisfaction and even a *tremendous* amount of stress [34,35]; they are 6 times more likely to be exposed to the risk of mental health illnesses than the general population [33]. We designed the chatbot conversation to concern the life of graduate students, instead of addressing all populations [36], to suit a more focused, contextualized conversation.

### Research Questions

This study aimed to design a conversational sequence for a brief motivational interview for stress management and to investigate its conversational experience with a group of graduate students on a Web-based text messaging application. The research questions were as follows:

- How do users perceive the sequenced conversation with regard to the MI components?
- What aspects of the conversation can help graduate students cope with stress?
- In what ways can the conversation be improved for better mental health support?

Our study shares much with the computerized MI dialogues in earlier research [13,14] yet differentiates itself in that it is designed for a potential mental health concern and that it uses a sequential organization of MI skills to investigate the user experience of an automated counseling conversation.

## Methods

We have illustrated how we translated the theoretical aspects of MI in a Web-based text messaging application called Bonobot. It generates an automated conversational sequence of a brief motivational interview with graduate students for coping with stress.

### Technical and Relational Components of Motivational Interviewing in Chatbot

MI entails technical and relational components [1]. The technical component includes counsellor techniques (eg, open questions and reflections) to facilitate change talk, where the client argument for behavior change is formulated [37,38]. The relational component [38], an empathic understanding experienced by clients as the counsellor helps them verbalize change, is also important to the efficacy of MI. In this study, the technical component is translated in a series of MI skills to represent MI counsellor behavior that may evoke change talk. As for the relational component, as Bonobot as a nonhuman agent is inherently incapable of empathy, we tried to achieve such a feat by designing its interaction in the following manner: (1) contextualizing the chatbot responses to the graduate school context [39]; (2) not bombarding questions at the user [29,40]; and (3) applying different combinations of MI skills [41] in the progress of communication.

Though a human MI counsellor would make use of MI-consistent skills spontaneously, a fully natural language conversation is a feat beyond current technology. We have worked around this problem by employing a summons-answer sequence, which can facilitate an exchange of volleys [41] between the summoner and the summoned. Here, the summoning agent asks questions to which the summoned user answers. The agent, in turn, gives feedback. Such an orderliness continues with alternations of volleys between the 2 parties, as in an *abab* formula [28].

### Formulating Chatbot Responses

To ensure Bonobot provides responses in appropriate MI skills and communicates them in a proper manner to qualify for both MI components, its responses took the following steps in preparation. First, SHP and JC collected model counsellor statements that may qualify for MI skills from the literature [24,42-48]. Second, they reviewed the statements to gather more generic ones. For example, they removed statements that are narrow-focused (eg, “You’ve been homeless since April...what happened that made your anger reach a breaking point last night?” [45]) and blanked portions of statements to be replaced with fillers from client input (eg, “What was helpful when you feel (client_input_emotion)?” [43]). Finally, to help the agent be more expressive of empathy with regard to the life of a graduate student, SHP and JC modified and added more contextualized statements (eg, “What were your initial goals when you first planned for a graduate degree?”).

A total of 220 prepared statements were later reviewed by certified therapists, CK and SL. They first considered the Motivational Interviewing Skills Code (MISC) [41] to evaluate the responses with regard to MI. As our study primarily concerns the chatbot responses as MI counsellor language, the therapists used the Motivational Interviewing Treatment Integrity (MITI) [49] that refers only to the therapist behavior [38] from the MISC. They coded each statement with the following MITI categories. Examples of the coded responses are provided in Table 1. For predefined (giving information [GI]) responses, see Multimedia Appendix 1.

- *Giving Information* *(GI)* *.* MI counsellor gives information to educate or provide feedback. As for Bonobot, it provides templated responses to address its role, privacy rules, and the beginning and closing of the session.
- *Questions (Q).* In MI, the counsellor is expected to ask questions that invite elaboration on the problem as well as questions that may evoke change talk. Bonobot uses both types according to the stage of the conversation: *focusing questions (FQ)* and *evoking questions (EQ)*.
- *Reflections (R).* Reflections convey understanding, facilitate exchanges, or further add substantial meaning to what clients say. Bonobot uses simple reflections to acknowledge client remarks and lead the conversation.
- *MI-Adherent Statements (MIA).* MI-adherent statements include any counsellor behavior that is aligned with the MI approach. For Bonobot, we intended affirming statements that may posit client traits in their articulating change.

### Sequencing in Chatbot Conversation

**Figure 1:**
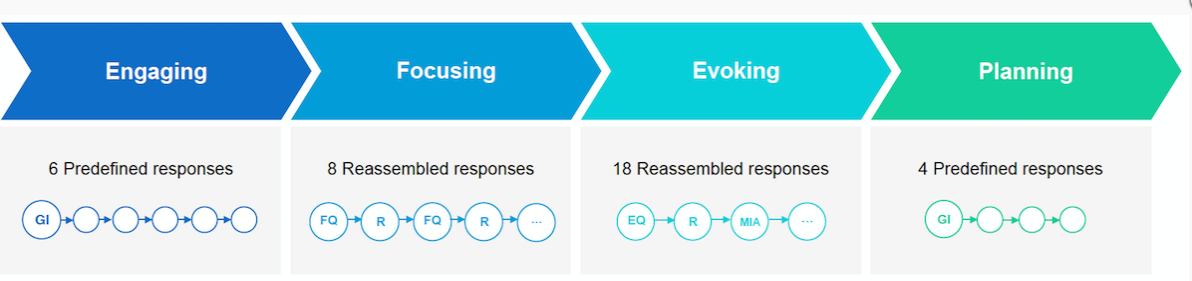
The stages and sequence of the Bonobot conversation. EQ: evoking questions; FQ: focusing questions; GI: giving information; MIA: motivational interviewing–adherent statements; R: reflections.

Bonobot is built to lead a structured conversation that follows the flow of an MI interaction, namely the 4 processes of MI [29]: Engaging, Focusing, Evoking, and Planning (see Figure 1). Engaging builds a relational foundation with the client. The client’s target behavior is determined in Focusing. In Evoking, change is explored, ideally with the resolution of ambivalence. Planning consolidates client commitment and actions. As Bonobot can only utilize predefined responses, we defined operational aims to reflect the 4 processes within the technical boundaries. In Engaging, Bonobot shares brief introductions with the user and gives instructions to use the chatbot. In Focusing, Bonobot asks the user to detail their problem, possibly having them identify an inner struggle. This leads to Evoking, where Bonobot explores future goals with the user, affirming their own ideas for change. Finally, Bonobot invites the user to ponder the overall session in Planning.

To address the aim of each process, Bonobot uses different combinations of MI skills in each stage (see Figure 1). For the first and last stages, Engaging and Planning, respectively, Bonobot interacts with predefined GI templates to properly manage the beginning and ending of the conversation. In Focusing, FQs are followed with Rs to reveal and reflect on any struggle about the problem. In Evoking, EQs are prompted to encourage change talk and are followed by Rs and MIAs to explore and affirm the idea of change. As advised by the literature [29,40], no more than 2 questions are asked in a row. Rs and MIAs are primarily placed after FQs and EQs as feedback.

**Table 1:** Examples of Bonobot responses by motivational interviewing (MI) skill.

MI skill	Questions type	Example response
Questions	Focusing questions	In what way does this bother you?
		How would you feel about that?
	Evoking questions	How have you coped with difficult times in the past?
		What were your initial goals when you first planned for a graduate degree?
Reflections	—^a^	It’s tough being a grad student.
		You certainly have a lot on your mind.
MI-adherent statements	—	Sometimes you show a determination that surprises even you.
		It seems like you are a really spirited and strong-willed person in a way.

^a^Not applicable.

### Implementation of the Chatbot Application

Bonobot runs a conversation by generating responses based on keywords. We extended the framework of ELIZA [50], the first chatbot in history, so that Bonobot identifies user keywords but generates responses in the form of an MI skill. We also built 2 modules in the application, Flow Manager and Response Generator, which would execute the sequence and assemble responses.

#### Preparing Keywords and Responses

A pool of keywords and responses was prepared in a script for Bonobot as shown below:

- *Keywords.* We replaced most keywords in a reproduced ELIZA script [51] with ones obtained from 2 online graduate student communities (r/PhD [52] and r/GradSchool [53]) on Reddit, a social media platform. SHP and SL categorized 1000 posts from each community by open coding for topics based on the title and content [54]. JC reviewed the topics and resolved disagreements with SHP. A word frequency analysis using tf-idf [55] yielded keywords by topic. SHP, JC, and SL went through an iterative process of distributing weights to keywords, so that the graduate school–related ones would be weighted higher. A total of 70 keyword categories were prepared.
- *Responses.* SL programmed responses to be reproduced from the pool of prepared MI statements, triggered by keywords from user input. For each keyword, a designated set of MI skills was allotted. Altogether, with repetition, a total of 209 FQs, 188 EQs, 166 Rs, and 140 MIAs were prepared in the chatbot script. There were 8 GI templates to be used in the beginning and end of the conversation. We also included extra responses to resume the conversation in case no keyword was matched. 

#### Running the Conversation

Bonobot’s 2 modules, Flow Manager and Response Generator, were programmed in JavaScript language. Python’s Flask 1.0.2 framework was used as the Web application server. The modules work together to run the 4-staged conversation. A series of pilot study sessions informed the final sequencing and turns.

**Figure 2:**
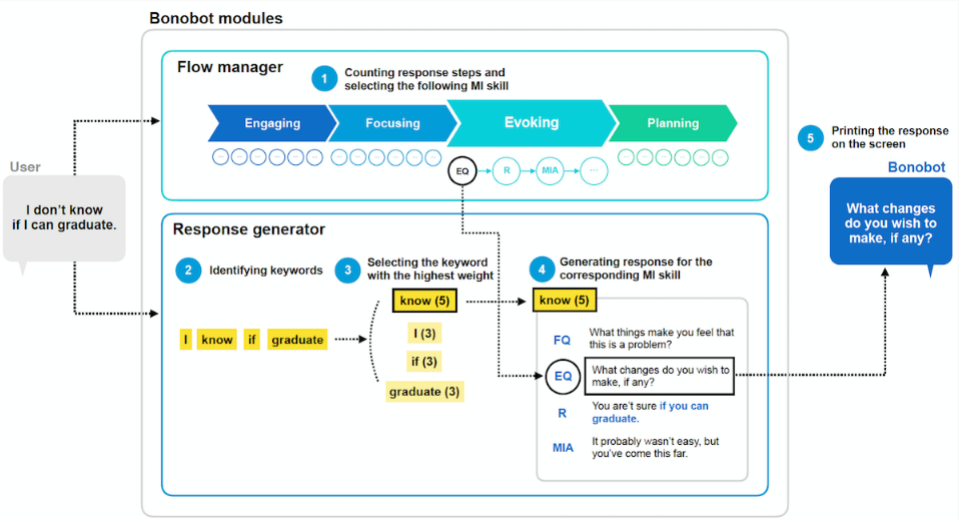
The automated generation of responses via Bonobot modules. EQ: evoking questions; FQ: focusing questions; MIA: motivational interviewing–adherent statements; R: reflections.

- *Flow Manager.* Flow Manager runs the conversation from one stage to another. At the beginning and end of the conversation, it assigns templated responses to lead the user into and wrap up the conversation. In between, Flow Manager counts the steps in a sequence so that the conversation follows the sequence. If a user does not respond in 10 seconds, it prompts an additional question from Response Generator.
- *Response Generator.* Response Generator identifies keywords and assembles responses (see Figure 2). For instance, suppose a user types in “I don’t know if I can graduate.” in the Evoking stage. Flow Manager alerts the MI skill to be printed next (“EQ”) and Response Generator extracts keywords from the user’s input (“I”, “know”, “if”, and “graduate”). It prints the reassembled response (“EQ”; “What changes do you wish to make, if any?”) under the highest weighted keyword (“know (5)”). It never repeats the same response twice.

**Figure 3:**
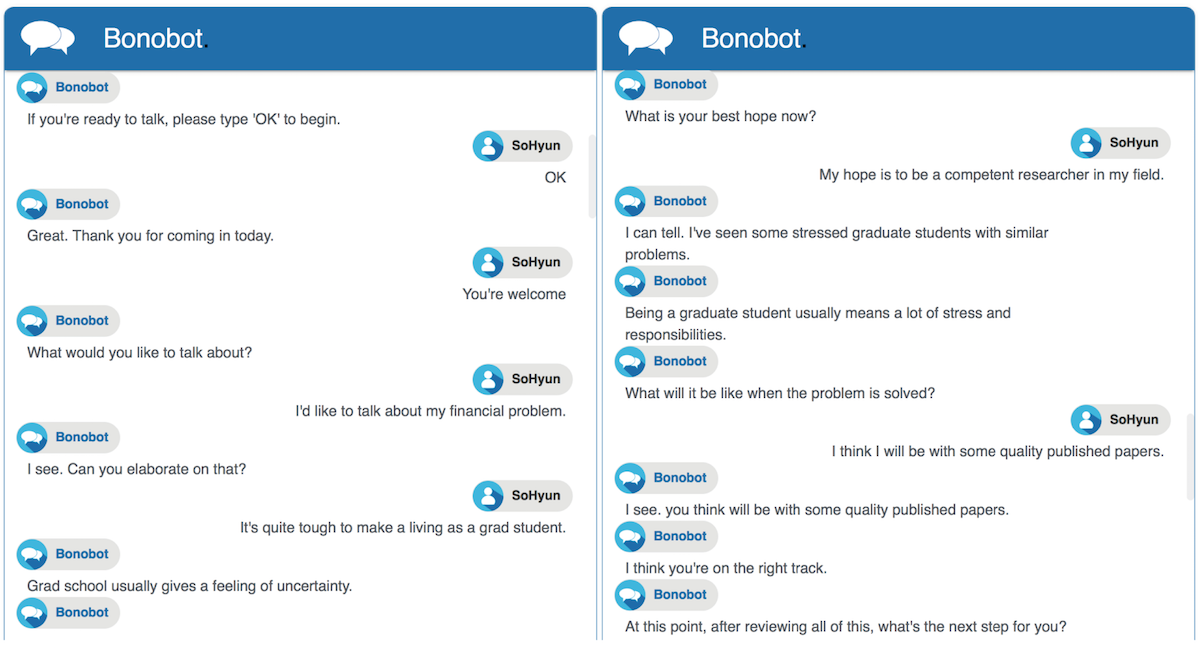
Excerpts from example conversations with Bonobot (For a full example, see Multimedia Appendix 2).

Pilot sessions with 10 graduate students (7 males) aged between 24 and 32 years determined 2 distinct sequences for the Focusing and Evoking stages: (1) to encourage the user to share the problem, an FQ is followed by an R; and (2) to affirm the user’s consideration of change, an EQ-R pair is followed by an MIA. In each stage, Bonobot is to repeat the sequence 4 and 6 times, respectively (see Figure 1). This will make up a total of 8 and 18 Bonobot turns in each, with possible extra ones due to the 10-second inactivity rule. Finally, the conversation takes place on a text messaging application in an internet browser (see Figure 3 and Multimedia Appendix 2).

### Study Design

A study was designed to investigate (1) the conversational user experience with Bonobot; (2) the impact on their coping with stress; and (3) their needs for better mental health support.

#### Task and Recruitment

A chatting session with Bonobot was prepared for study participants. An advertisement for volunteers was posted on a Seoul National University online bulletin. A total of 30 full-time graduate students were recruited. The inclusion criteria were that they could (1) communicate with the chatbot in English, (2) share their concerns about school, and (3) participate in an interview about the chatting experience.

#### Procedure

Participants were invited into a room with a comfortable chair, big table, and laptop computer. A laptop was used instead of the user’s mobile phone for consistency and screen convenience. After SHP and JC gave a brief introduction, participants answered a survey of demographic information and the Perceived Stress Scale (PSS-10) [56]. After SHP and JC explained how to use the chatbot, they exited the room for the participants to chat with Bonobot alone. They returned on the participants’ notice and conducted semistructured interviews, reviewing the conversation on the laptop screen. The entire process was designed for an hour, and participants received a US $10 beverage coupon as a reward upon completion.

#### Ethics Approval

Before they gave consent, all participants were informed of the purpose and procedure of the study and that they could resign from it at any point if they felt uncomfortable. The study conformed to the principles of scientific research with human subjects. It was approved by the Seoul National University Institutional Review Board (IRB No. 1708/001-018).

#### Data Collection and Analysis

In this study, 2 types of data were collected. First, participants’ age group, gender, and perceived stress scores were entered on Microsoft Excel by SHP. Perceived stress was measured on a 4-point Likert scale. PSS-10 is one of the most widely used instruments to assess one’s perception of stress in the course of the previous month, and higher PSS scores are associated with higher risks to negative health conditions [56]. The collected scores were computed for mean and SD values.

SHP and JC conducted semistructured interviews as student project coordinators and collected the data. They inquired (1) any sequential encounter that went particularly well or oddly and why; (2) whether the conversation had any impact on their perceived stress or not and in what ways; and (3) any suggestions for better mental health support. Detailed questions were asked ad hoc by SHP for an in-depth elaboration on the conversational experience, and notes were taken by JC to record participant-indicated conversational happenings. The interview was designed for 30 min and did not exceed 40 min at most. All interviews were audio-recorded with the consent of the participants and anonymized with numbers.

Interviews were analyzed via a 6-phase process of thematic method by Braun and Clarke [54]. First, all interviews were transcribed verbatim by JC on Microsoft Excel. SHP reviewed and segmented the transcripts by each anonymized participant, using Optimal Workshop’s Reframer tool [57]. SHP and JC went through a process of open coding the segments to generate initial codes by tagging each with free-phrased labels. Labels were reviewed and renamed for initial codes. Codes were once again reviewed to search and define themes. As advised by Braun and Clarke [54], themes that merely reiterate the interview questions were avoided but those that can reveal the depth of the data were reviewed and redefined in iteration. Finally, 3 pairs of themes were prepared: Evocative Questions and Unfitting Feedback; Invoking Self-Reflection and Encouragements for Graduate Students; and Need for Informational Support and More Contextualized Feedback. In reporting the results, we rephrased the pairs into 3 overarching themes to answer each research question.

## Results

### Participant Demographics

Participants were in their 20s (n=20) and 30s (n=10), and a half of them were male (n=15). The average PSS score was 22.5 (SD 5.0), higher than the norm [56]. Conversation topics included the lack of confidence in academic work and research (40%, 12/30), psychological burden of writing theses (17%, 5/30), financial constraints (10%, 3/30), uncertainty about the future (10%, 3/30), work-life balance (7%, 2/30), people skills (7%, 2/30), and other (10%, 3/30). These topics of concern were mostly in agreement (90%, 27/30) with the themes discovered from the content analysis from the Reddit posts.

### Qualitative Findings

From the conversation, participants preferred Bonobot’s questions to its feedback. EQs were a good means of reflecting on themselves and for some, an instrument for motivational boost. Some of Bonobot’s responses related to graduate school were appreciated. All participants favored the idea of using a chatbot for coping with stress, with suggestions for better support. The themes are detailed below.

#### Evocative Questions and Clichéd Feedback

Participants mostly favored the way Bonobot kept asking them questions. It felt like they were being heard (n=18). In particular, they preferred the EQs in the third stage as they were “something new and interesting” (P2) and “triggered inspiring ideas” (P13). P1 said he liked them as “the questions were profound [...] I had to think deep down and discover the answers inside.” Questions such as “What can be some of the good things about making a change?”, “What do you wish to be different?”, “How would you like things to turn out for you?”, and “What could be the next step now?” triggered to think “who you really are and what you really want” (P12) and “what needs to be done to achieve your goals” (P13). P11 said that “it was really the third stage” that “felt quite convenient to draw something out” from him.

However, they did not like questions that made them reiterate their answers. Although Bonobot did not ask the same question twice, participants felt that some questions had them elaborate themselves again. This was a bit annoying to some participants (n=7). In addition, some questions did not feel productive when they were not relevant to the context of their problems and spanned too grand a scheme of things. P4 pointed out an example:

Bonobot asked me what I would have chosen to do if I did not pursue a graduate degree. But I’ve never thought of such an idea—something other than grad school. I’d say that wasn’t quite helpful.

P30 added that “the questions aimed too broad a range that each question could entail a whole lot different story by itself. I think the conversation was too short for that.”

In between the questions, Bonobot gave feedback that intended reflections and affirming statements. Most participants (n=21) liked Bonobot saying “such sweet words” (P21). P9 said, “I thought Bonobot used words of empathy really well, you know, even if some felt like templates, they were good.” However, it was not quite up to their expectations (n=13). P29 said, “here, Bonobot said the right thing, but it doesn’t fit into what I said. I had to doubt whether it really understood me.” P2 also said that “I know it tried to encourage me, but sometimes it did at the wrong time, which made me wonder if Bonobot was to encourage me no matter what.” P10, P16, and P25 said they anticipated something more than Bonobot simply repeating what they have said. P27 recalled, “It was not bad, but it can be weird… you don’t really recite word for word when you talk.”

Some feedback, such as “I hear your struggle.”, “You certainly have a lot on your mind.”, “That’s understandable.”, and “You’re not the only one in this.” also felt rather banal. P4 said “Some felt like they were just there because they had to.” P21 and P24 found it odd that Bonobot repeated similar expressions in the conversation. In addition, they were just “too nice” (P2). P1 added that “you know, if you were to talk with a human being, you wouldn’t really say the nicest things throughout.” P4 still “appreciated the niceness” as he rarely has a chance for those words. However, for P17, P20, P24, and P28, words of empathy only echoed what they could expect from anybody around them.

#### Self-Reflection and Potential Consolation

Most found the Evoking stage an opportunity to reflect on themselves (n=19). P4 said “I think it was a time to reflect on me and my situation. [...] I liked I had the chance to rediscover myself with my own words by chatting with Bonobot.” For P22, it was “a Socratic method”: “In the end, you answer for yourself. Bonobot asks me questions, and by answering them, I get a better understanding of myself.” P23 also said it was like “a catalyst” that kept nudging her to think about herself and her life. This self-reflection spurred a sense of motivation (n=11) that was “sort of buried in” (P27). P30 said she could gain a motivational boost:  

You know, I’m always like, “what am I to do now?,” “this is too hard,” and “I can’t do it.” Now, I have this question inside, “so how do I want this to be resolved?” This can move me forward. I feel like I need to do something about it.

The progression toward Evoking, however, was not everyone’s preference (n=6). P17 said, “Bonobot clearly had an idea about what it wanted to hear from me—something positive—and it wanted me to say it, which made me feel like Bonobot had the lead over the conversation, not me.” P18 also disliked the idea of “having a conversation with a purpose.” P20, in particular, had trouble facing the idea of change:

I guess I am not exactly sure. I know I need to change, and I know what I need to do to make that change happen. But that’s causing the stress! But Bonobot’s questions felt like it was trying to remind me of that, instead of letting me vent.

Participants appreciated Bonobot taking the role of a nonjudgmental listener (n=11). P3 thought she made “a virtual friend who listens to [her] and tries to understand [her].” It was essentially a private conversation where they could talk about things that they cannot usually open up to their family or friends (n=8). P19 said he could feel more relaxed talking to Bonobot “for [he] did not have to worry about what Bonobot would think of [him].” P24 said he shared the same subject that he did with a colleague, which ended up in an argument. He felt better talking with Bonobot “for [Bonobot] does not have any interests that may conflict with mine.”

They preferred Bonobot’s words of empathy that concerned the life in graduate school (n=13). P3 said that Bonobot seemed to know “what it is to be a graduate student.” P6 felt touched when it said, “Don’t let it discourage you,” to his disconsolation with his progress. When Bonobot asked P7 about the past achievements to which he had none, it replied “That’s okay,” with which he felt so moved and said, “it understood me like a human being!” P23, in particular, was pleased with Bonobot saying “A lot of graduate students suffer from variations of the same problem.”, when she confessed trouble with her advisor. Similarly, P15’s favorite was “That’s exactly how many students feel during their graduate program.” P9, P19, and P30 also indicated the following as the best responses: “Grad school usually gives a feeling of uncertainty.” and “It’s tough being a grad student.”

#### Need for Information and Contextualized Feedback

Talking about making a change brought about a need for data and information (n=9). P7 said “I need more information I guess, about the problem I talked about.” P8 said the conversation would have helped “if the chatbot offered tips for writing theses on the Internet.” P26 suggested the following:

a chatbot can deliver news articles or life tips. You know, say I have a sleeping problem. It can give me suggestions, such as music recommendations, health information, or other tips found online.

Some participants also indicated a need for making plans for change (n=5). P13 recommended that Bonobot ask more detailed, branching questions such as “How much financial aid do you get?” or “What are the current career options?”. P5 said “I would appreciate it much more if it organized for me a listed reminder of the things that were brought up in the conversation.” P14 and P22 also suggested that discussing specific action items would be helpful. P26 said that planning an agenda with Bonobot would potentially inspire a sense of partnership.

Most participants said they would prefer having an in-depth conversation, digging deeper a single subject instead of a range of things (n=17). P9 thought the conversation tried to cover too much: 

if I were to talk about the same subject with my friend, we would talk about it for a long time. But in this conversation, it feels like I need to share just enough and then move on right away to “think about the next, and the next.” I wish it would rather ask me more details about my problem.

Other participants also agreed that they would want a chatbot to ask them more specific questions about their life, “as if [they were] talking to a human being” (P20). Addressing such an elaborated context of their problems would signal “a continued relationship” (P5) with the chatbot (n=7).

In addition, participants wanted more emotional responses that are appropriately contextualized to their input (n=13). P12 said, “This chatbot can say some sweet words, but I would prefer more emotional expressions like, ‘I can’t believe that happened to you!’ or ‘That must have been very hard on you,’ things like that.” P27 put it this way: “You know, I’d like words that are more for me and me only, not like the mundane ones that anybody can say to everyone else.” For P25, more personalized responses would have helped her feel more empathized:

What if it said something more concrete, like, “You must have had a hard time communicating with your advisor all this while,” instead of just a simple expression of empathy? Then I would think that it really understands my feelings.

## Discussion

### Principal Findings

This case study has explored conversational experience of an automated sequence of MI skills for stress management on a Web-based text messaging application, with an aim to integrate both technical and relational components of MI. Participants found the sequenced conversation quite natural and easy to grasp and follow. The evocative questions inspired discussions about change for most, encouraging self-reflection. Some agent-generated feedback that reflected their life in school was appreciated. Participants suggested informational support for the chatbot to equip a better preparation tool for helping change. A need for an in-depth conversation was also indicated, with more contextualized feedback for better engagement.

Our study highlights the possibility of applying a sequential approach to constructing an automated motivational interview with MI skills to integrate both technical and relational components. Previous research has remained rather vague in explicating the relational component or has excluded it in its entirety owing to technological issues (ie, [1,12]). It is important that the MI counsellor uses interactional skills strategically to convey an empathic understanding [15]. However, not much has been studied on how one can translate such a technique in a computational manner. We designed a structured conversation with a sequence of MI skills, with an effort to incorporate both components of MI. Using the summons-answer sequence [28], we placed questions sparingly and not consecutively [29] and assigned reflections and MI-adherent statements in-between to form the basis for an empathic understanding [39,41]. The result was the FQ-R and EQ-R-MIA sequences in the second and third stages, respectively, with GI templates at the beginning and at the end.

The sequence demonstrated a reasonable, though not optimal, MI interaction. The questions conveniently had the user talk about their problems, and the conversation encouraged a chance for self-reflection for most and inspired an idea of change for some participants. However, agent-generated reflections and MI-adherent statements received mixed opinions. Owing to its technical constraints as a chatbot counsellor, some of Bonobot’s responses were ill-assembled and could not correctly reflect or affirm on user volleys. Although participants liked the moral support from the chatbot’s feedback, we caution that it may risk sustaining the status quo instead of change [58]. An accurate empathy requires a profound contextual understanding, which is hardly achieved by nonhuman agents. Further research is needed to generate chatbot responses that are appropriately tailored as well as MI-consistent to avoid naively echoing client remarks in reflections and simply abstracting them in questions.

Participants regarded evocative questions as a constructive means to revisit their source of stress, leading to the idea of change. In the interview, participants who were able to ponder change were willing to share their immediate plans to cope. However, for some, the distaste and even resistance to problem-solving actions was also observed. We find both types of reactions to be in alignment with the literature [38], and highlight the potential influence of change talk on stress coping behavior. The Evoking stage could encourage self-reflection, potentially playing a part in coping with stress. It prompted participants to think why the problem is stressful and how they want it to be resolved. In terms of Lazarus and Folkman’s transactional model [59], this process is likely a cognitive *reappraisal* of the stressful condition. Such a positive reinterpretation is not only a means to reduce emotional distress but also a form of active, problem-focused coping [60]. This finding leads to a future research agenda to collect concrete evidence of change talk in chatbot-client conversations and measure its empirical effect on stress reduction as a coping intervention.

What participants requested for better mental health support suggests the potential of a chatbot counsellor, as well as milestones to be achieved in technology. Our study has made a meaningful step forward to address both components of MI in a chatbot app, supporting a real-time speaker exchange while utilizing different MI skills. As for the technical component, the results show that MI techniques were in action. Participants needed more informational support as they revisited their problem and began planning to cope. The chatbot app needs to be equipped with problem-related information in the future. In terms of the relational component, participants agreed on Bonobot’s caring attitude, a ground hypothesis for a client-centered approach [61]. However, the need for better contextualized feedback demands much advance in technology to generate intelligent, context-aware chatbot responses that can contribute to client change talk. Although the chatbot in our study could not fully exhaust the MI counsellorship, future research can make use of advanced algorithms such as artificial neural networks to generate more sophisticated chatbot responses that better reflect the theory and principles.

Our findings lead us to suggest that, if properly and carefully designed, a chatbot may conveniently serve the purpose of MI as an *interactional* practice in health [62,63]. As a real-time messaging application, chatbots can help tend communication for a therapeutic encounter between a counsellor and client. The recent chatbot apps that provide therapy (eg, [30-32]) mainly serve the role of delivering various treatment programs via a conversation. Our study stresses on the conversation with the chatbot itself as the potential medium to render a motivational interview, for mental health concerns in particular. As we face an unprecedently technology-intensive era, we foresee a number of conversational agents to appear in the communicative process of providing care (eg, [64-68]). To properly manage such an interaction, we believe a well-designed conversational sequence is necessary. We suggest having a close collaboration with human-computer interaction in studying the internet and mobile health care technologies [69] to address many complex problems that may arise with users verbally engaging in the technologically adapted treatment.

#### Limitations and Directions for Future Research

Our work is bound with limitations. First, Bonobot utilized 1 possible sequence of MI skills. A gamut of sequences can be tried for further investigation. In addition, there are a number of factors that may have contributed to the potential impact of the conversation, for example, the number of chatbot responses and order of skills to name just a few. More sophisticated designs of MI conversations can be explored in the future.

Moreover, Bonobot could only support a single session. We believe evocative questions made a positive impression for many to begin with. However, it could not be fully explored, along with the ambivalence associated with it, owing to technical and circumstantial constraints. A continued, fully fledged MI session should follow to investigate the longitudinal effect of change talk as a coping instrument.

As a case study, our research presents a small participant sample, and hence, the scope and range of stressors of graduate students could not be exhausted. A stressor can be a complex, multi-layered problem, spanning various aspects of one’s life. Setting the scope of a stressful state context would be an important challenge to be resolved in the future, before a larger field study can be conducted.

This study also lacks a quantifiable measure in assessing the chatbot conversation. Although there are great inventories for evaluating client-counsellor or doctor-patient relationships (eg, [70,71]), few exist for human-agent interaction in health care encounters. Future research, possibly in human-robot interaction, could address this need for a toolkit to assess client-chatbot conversations.

Finally, other types of multimedia, in the form of animated characters, robots, avatars, or other embodiments, were suggested by a few participants for more affability and sociability (P5, P20, and P23). Our research only allowed text-based communication, even without textual emojis, to control for any effect other than from the sequence. It would be interesting to explore the effectiveness of multimedia resources or embodiment features on the relational component of MI.

### Conclusions

This case study has designed a conversational sequence of MI skills for an automated motivational interview on stress management and presented qualitative feedback from 30 participants on their conversational experience. Our findings revealed user preference for evocative questions but less inclination for agent-generated reflective and affirming feedback. Sequencing questions and MI-adherent statements can lead a conversation for coping with stress, possibly encouraging self-reflection. Participants demanded informational support as well as more contextualized words of empathy. Our study contributes to technological adaptations of MI and informs the design of future conversational agents in mental health care.

## Appendix

Multimedia Appendix 1 Bonobot responses by motivational interviewing skill.

Multimedia Appendix 2 Example conversation with Bonobot.

## Abbreviations

EQ: evoking questions
FQ: focusing questions
GI: giving information
MI: motivational interviewing
MIA: MI-adherent statements
MISC: Motivational Interviewing Skills Code
MITI: Motivational Interviewing Treatment Integrity
PSS: Perceived Stress Scale
Q: questions
R: reflections
