# Supplementary material for: Designing a Chatbot for a Brief Motivational Interview on Stress Management: Qualitative Case Study
Source: J Med Internet Res. 2019 Apr 16;21(4):e12231. doi: 10.2196/12231 (PMC6488959; doi:10.2196/12231)
Supplement: Multimedia Appendix 1 [file jmir_v21i4e12231_app1.pdf]

List of Model Chatbot Responses by MI Skill

No.	MITI Code	Q Type	Statement
1	Q		Hi #(username). I’d love to hear what that’s all about. Do you have any concerns you'd like to talk with me?
2	Q		Hello #(username). What brings you in today?
3	Q		Hi #(User_name). I know you had some things you wanted to talk about, so what brings you in today?
4	Q		I assume, from the fact that you are here, that you have some things you want to talk over with a counsellor. What would you like to discuss?
5	Q		I'd like to understand how you see things. What's brought you here? What's been the problem?
6	R		You have struggled so much inside yourself that you weren't sure how to be open about that struggle.
7	I		I am a chatbot that listens to your stories and helps you guide yourself.
8	Q	FQ	You sometimes wonder who could possibly understand all that you've been through and what this has meant for you as a person. Would you share some of your struggles with me?
9	I		Since I respect your privacy seriously, you feel free to share whatever you would like.
10	I		Since I take your privacy seriously, you can talk about anything you would like, without fear of that information leaving the room.
11	I		Since I take your personal information seriously, I hope you feel comfortable sharing your feelings, concerns, and problems.
12	I		Everything you say in this chat will be kept secret, so no worries!
13	I		I won't disclose personal information about your feelings, their pasts, and concerns.
14	Q	FQ	In what way does this bother you?
15	Q	FQ	In what ways does this make sense to you?
16	Q	FQ	What things make you think that this is a problem?
17	Q	FQ	What difficulties have you had in relation to your problem?
18	Q	FQ	What worries you about your *(problem)?
19	Q	FQ	In what ways do you think you have been harmed?
20	Q	FQ	What is it like for you to share what you've been keeping inside for so long?
21	Q	FQ	What keeps this problem going?
22	Q	FQ	How much does that concern you? In what ways?
23	Q	FQ	How would you feel about that?
24	Q	FQ	How has *(problem/issue) affected your daily routine?
25	Q	FQ	How long has it been going on?
26	Q	FQ	How has your *(problem) stopped you from doing what you want to do ?
27	Q	FQ	Anything specific about your thought that made you feel as you do?
28	Q	FQ	What made you nervous?
29	Q	FQ	What contributed to this nervousness?
30	Q	FQ	What kinds of things can help you settle down?
31	Q	FQ	What can you do about your frustrations?
32	Q	FQ	What steps can you take to avoid the frustrations?
33	Q	FQ	What methods do you currently use to calm your self talk and in what ways are they not working for you?
34	Q	FQ	Where do you think your *(feeling) about being still stems from?
35	Q	FQ	What have you done to cope with these feelings?
36	Q	FQ	What will make you feel better?
37	Q	FQ	What can you do to help decrease your stress?
38	Q	FQ	How do you feel not being the high achiever that you were before at school?
39	Q	FQ	When did this problem begin?
40	Q	FQ	How did you come to that conclusion?
41	Q	EQ	What worries you about your current situation?
42	Q	EQ	What concerns you the most about this in the long term?
43	Q	EQ	What do you think will happen if you do not take action against your problem?

44	Q	EQ	In what ways does this concern you?
45	Q	EQ	What do you imagine are the worst things that might happen to you *(physically, psychologically, socially)?
46	Q	EQ	What is there about your problem that you or other people might see as reasons for concern?
47	Q	EQ	What concerns you the most?
48	Q	EQ	What are your worst fears about what might happen if you don't make a change?
49	Q	EQ	What would your friends say about *(problem/your behavior)?
50	Q	EQ	What would your partner say about what you're doing?
51	Q	EQ	Tell me what your family would say about *(current behavior/problem)?
52	Q	EQ	If a friend was with you, would they see it the same way?
53	R		If I heard you correctly, this is what I think you are saying: *.
54	R		Given what you said, you might feel * (feeling).
55	R		You are having trouble with *(problem).
56	R		Some of what I heard you say *.
57	R		You seem to be thinking that you might be able to do * or *.
58	R		You're disappointed that *.
59	R		You feel you have failed *.
60	R		It sounds like you may be feeling some * with that.
61	R		You sound *(emotion) with *(problem).
62	R		You feel *(emotion) because you cannot *(problem) and you want to *(goal).
63	R		You are frustrated about not being able to have better control *(problem) despite doing your best.
64	R		You are not sure what else to do.
65	R		You are struggling with figuring out what else you could do to improve *(problem).
66	R		You care so much about controlling your *(problem) and you want to figure out what else you could work on changing to help you better control it and live better life.
67	R		You are certainly having to cope with a lot of problems right now-more than most people. I can understand how sometimes you want a "lift" so badly, you want a release from it all.
68	R		On one hand, you feel you have done all you could do to control your *(problem), but on the other hand, you want to figure out what else you need to do.
69	R		You are feeling overwhelmed with work.
70	R		You certainly have a lot on your mind.
71	R		You are not sure how anyone can help.
72	R		You are the kind of person that follows through when you set your mind to something.
73	R		You might feel little angry at yourself, for getting into this situation.
74	R		You're willing to take responsibility for your situation and life.
75	R		I somehow sense that you feel *.
76	R		You feel overwhelmed, and very, very tired, even with things that you used to be able to manage.
77	R		I appreciate your willingness to discuss possible options.
78	R		You seem to be unsure of how to deal with this problem, which leaves you feeling helpless, sad and hopeless.
79	R		It sounds like you are overloaded and anxious. You feel bogged down.
80	R		I am guessing it was very hard and difficult for you to endure this situation.
81	R		I see this is really bringing up some sore spots for you.
82	R		I can feel you. I have seen a lot of overly stressed students like you due to similar problems.
83	R		Everyone gets mad sometimes; it's not just you.
84	R		Nobody can promise you that things will be easy but you will get better.
85	R		That's understandable.
86	R		It is understandable that you feel anxious in this situation.
87	R		It sounds like you had a rough semester.
88	Q	FQ	What else?
89	Q	FQ	What else can you think of?
90	Q	FQ	It's understandable. Please continue.

91	Q	FQ	What happened next?
92	Q	FQ	What are your thoughts regarding *(problem)?
93	Q	FQ	What are you thinking about at this point?
94	Q	FQ	What do you mean?
95	Q	FQ	Please go on.
96	Q	FQ	Can you elaborate on that?
97	Q	FQ	Give me an example.
98	Q	FQ	What other problems have you had?
99	Q	FQ	I understand that you have some concerns about *(problem). Tell me more about them.
100	Q	FQ	Is there any other context that you would like me to understand?
101	Q	FQ	Could you give me some of the details?
102	Q	FQ	Help me better understand *(problem), and how it has affected you life.
103	Q	FQ	Please go on. I am with you.
104	Q	FQ	Could you say some more about that?
105	Q	FQ	That's helpful. I'd appreciate if you could give me more detail.
106	Q	FQ	I'm beginning to get the picture: but some more examples might help.
107	Q	EQ	What do you say to yourself that helps you cope?
108	Q	EQ	What do you do that helps?
109	Q	EQ	What supports do you draw upon?
110	Q	EQ	What was helpful when you feel *(feeling)?
111	Q	EQ	What strong points do you have that could help you succeed in making this change?
112	Q	EQ	How have you managed with all you have been through?
113	Q	EQ	What supports do you draw on in your institution?
114	Q	EQ	What supports do you draw on in your family, friends, or neighborhood?
115	Q	EQ	What sources of social support do you have?
116	Q	EQ	Is there someone at your institution can help you with this?
117	Q	EQ	Are there others you could call for support? In waht ways?
118	Q	EQ	Who else could help with change?
119	Q	EQ	When in your life have you made up your mind to do something, and did it?<br>It might be something new you learned, a habit that you quit, or some other significant change that you made in your life.<br>What did you do that worked? What obstacles were there, and how did you surmount them?
120	Q	EQ	What did this mean about your resources, skills, and strengths?
121	Q	EQ	What do you say to yourself that helps you cope?
122	Q	EQ	What strong points do you have that could help you succeed in making this change?
123	Q	EQ	Think about times in the past that you have been able to catch yourself like this. How were you at that time?
124	Q	EQ	What is there about you, what strong points do you have that could help you succeed in dealing with this kind of problem?
125	Q	EQ	Strong and loving, smart, persistent. Sounds like you have a lots of what it takes to handle tough changes.<br>How about this? Give me and example of a time when you really wanted something and you went after it.
126	Q	EQ	What might you do if...?
127	Q	EQ	How could you respond if...?
128	Q	EQ	What do you think would happen if...?
129	Q	EQ	How have you coped with difficult times in the past?
130	Q	EQ	You may have felt this way before. Feelings that felt really tough to deal with at that time, turned out to be temporary. How did you cope with it then?
131	Q	EQ	Do you remember a time when things were going well for you? What has changed?
132	Q	EQ	What were things like before you face * (problem)? What were you like back then?
133	Q	EQ	How has *(problem) stopped you from growing, from moving forward?
134	Q	EQ	What's different about the times when you don't have this problem?

135	Q	EQ	If you decide to make a change, what are your hopes for the future?
136	Q	EQ	How would you like things things to turn out for you?
137	Q	EQ	I can see that you are feeling really frustrated right now. How would you like things to be different?
138	Q	EQ	What are the options for you now? What could you do?
139	Q	EQ	If I waved a magic wand over your head and the problem that brought you here has been solved. What will you notice will be different in your life?
140	Q	EQ	Suppose that you did succeed and are looking back on it now: What most likely is it that worked? How did it happen?
141	Q	EQ	Suppose that this one big obstacle weren't there. If that obstacle were removed, then how might you go about making this change?
142	Q	EQ	Clearly you are feeling very discouraged, even demoralized about this. So use your imagination: if you were to try again, what might be the best way to try?
143	M		I appreciate how hard it must have been for you to decide to come here. You took a big step!
144	M		I think it's great that you want to do something about this problem. That must have been very difficult for you.
145	M		You are certainy a resourceful person, to have been able to live with the problem this long and not fall apart.
146	M		That's a good suggetion.
147	M		It must be difficult for you to accpet a day-to-day life so full of stress. I must say, if I were in your position, I would also find that difficult.
148	M		It seems like you are a really spirited and strong-willed person in a way.
149	M		Somehow, you've been able to meet the challenges that were once so frightening to you.
150	M		Sometime you show a deteremination that surprises even you.
151	M		You've presevered, despite circumstances that many others would not have been able to meet head on as you have.
152	M		You have shared so many strong feelings with me.
153	M		Thank you for sharing your struggles with me.
154	M		I appreciate your willingness to share so openly about your *(problem).
155	M		You have the courage and tenacity to overcome any challenge you face.
156	M		With all the obstacles you have right now, it’s *(impressive, amazing) that you’ve been able to refrain from engaging in *(risky/problem behavior).
157	M		I must say, if I were in your position, I might have a hard time dealing with that amount of stress.
158	M		You showed a lot of *(strength, courage, determination) by disclosing your problem.
159	M		I think what you are doing is really difficult. I'm really proud to be working with you on this.
160	M		If I were in your shoes, I don’t know if I could have managed nearly so well.
161	M		You have worked hard to do the right thing, despite how hard it is.
162	M		You have been successful in sharing your thoughts and being truthful about your emotions.
163	M		You feel proud of yourself for being able to control your *(problem).
164	M		You have had some significant setbacks in your life.
165	M		Somehow, you have been able to meet the challenges that were once so frightening to you.
166	M		You endured that even though you were under all that pressure? That's fantastic because it would have been easy to give up and you didn't.
167	M		I have heard a lot of students say that.
168	M		That’s a pretty common reaction.
169	M		Many students struggle with the same confusion about how to present the ideas in their mind.
170	M		Anyone would feel this way if they were in your situation.
171	M		I hear your struggle and I don't want to invalidate or minimize your experience but that's exactly how many students feel during their (graduate) program.
172	M		You're not the only one in this.
173	M		I would have felt that way too.
174	M		Based on what you’ve been through with your recent disappointing experience, it's no wonder that you’re ready to give up.<br>However, you still try to make an effort to make better your situation.
175	M		The emotional state you are experiencing and describing seems to me to be a normal response to your situation.
176	M		I’m impressed that you are still trying to improve your situation in spite of your frustration.
177	Q	EQ	How might you go about making this change?
178	Q	EQ	What would be a good first step?
179	Q	EQ	How will you go about it?
180	Q	EQ	What obstacles do you foresee, and how might you deal with them?

181	Q	EQ	What give you some confidence that you can do this?
182	Q	EQ	What do you think you will do ? What are you thinking at this point about *(problem)?
183	Q	EQ	What changes, if any, are you thinking about making?
184	Q	EQ	What can be some of the good things about making a change?
185	Q	EQ	At this point, after reviewing all of this, what's the next step for you?
186	Q	EQ	What could you do? What are your opions?
187	Q	EQ	It sounds like things can't stay the way they are now. What do you think you might do ?
188	Q	EQ	Of the things we have talked about, which ones concern you the most? What do you want to do at this point?
189	Q	EQ	Where do we go from here?
190	Q	EQ	What could be the next step now?
191	Q	EQ	How would you like things to turn out for you now, ideally?
192	Q	EQ	What do you wish to be different?
193	Q	EQ	What would be some of the good things about making a change?
194	Q	EQ	What will it be like when the problem is solved?
195	Q	EQ	What will you be doing instead?
196	Q	EQ	What else needs to happen to move you a little more towards your wish?
197	Q	EQ	How does this behavior fit into your value?
198	Q	EQ	What do you feel deep down in your heart about these dilemmas, or options?
199	Q	EQ	What is your best hope now?
200	Q	EQ	What are the most successful things have you accomplished? How did you plan to achieve that?
201	Q	EQ	What do you think you can do to manage all your responsibilities?
202	Q	EQ	How is going to school benefiting you?
203	Q	EQ	Do you think the decision on continuing with this loaded schedule will affect you in the near feature not only with grades, but also with your health?
204	Q	EQ	What do you think you can do next time before class to prepare you so you won't get bored in class?
205	Q	EQ	What are your goals now that you've started school and got your classes situated?
206	M		I certainly don't have the answers for you, but I have a lot of confidence that you do, and that working together we can find a way out.
207	M		You are the best judge what will work for you.
208	M		You are the best judge of what information is useful to you.
209	M		You are a free person, and it’s your choice. I couldn’t make the devision for you even if I wanted to.
210	M		I will be happy give you some ideas, but I don’t want to get in the way of your own creative thinking, and you are the expert on you.
211	M		I am not sure you really want my advice. Maybe you have some idea of your own about what to do.
212	M		Of course I can tell you what I think, if you really want to know. But I don’t want you to feel like I am telling you what you have to do.
213	M		I am not here to tell you what to do, or to try to make you do anything you don't wish to do. You will decide when and if you wish to change anything.
214	M		So, stick to what you know. And if you don’t know, see if you can find out. It often helps to get someone else's perspective on such issues.
215	I		You know, they say action is the foundational key to success. So keep it up!
216	I		They say life is trying things to see if they work. So let's not give it up!
217	I		It does not matter how slowly you go as long as you don't stop. We'll keep carrying on.
218	I		People say an obstacle is often a stepping stone. Don't let it discourage you.
219	I		Oops, but the time's almost up. We've got to wrap up our conversation today.
220	Q		So we talked about *(user keyword) today. How do you feel after talking about it now?

*( ) denotes placeholders for chatbot-generated replacements.
