# Supplementary material for: Designing a Chatbot for a Brief Motivational Interview on Stress Management: Qualitative Case Study
Source: J Med Internet Res. 2019 Apr 16;21(4):e12231. doi: 10.2196/12231 (PMC6488959; doi:10.2196/12231)
Supplement: Multimedia Appendix 2 [file jmir_v21i4e12231_app2.pdf]

Bonobot.

Bonobot

Welcome! What is your name?

SoHyun

SoHyun

Bonobot

Hi SoHyun! I'm BonoBot.

SoHyun

Hi Bonobot!

Bonobot

I am a chatbot that listens to your stories and helps you guide yourself.

SoHyun

That's cool!

Bonobot

Everything you say in this chat will be kept secret, so no worries!

SoHyun

Okay, I got it!

Bonobot

If you're ready to talk, please type 'OK' to begin.

SoHyun

OK

Bonobot

Great. Thank you for coming in today.

SoHyun

You're welcome!

Bonobot

What would you like to talk about?

SoHyun

I would like to talk about my graduate school life.

Bonobot

I see. Can you elaborate on that?

SoHyun

I don't know how I can manage my workload.

Bonobot

You don't know how you can manage your workload.

Bonobot

What things make you feel that this is a problem?

SoHyun

I think I have too many things to do and catch up at once.

Bonobot

Right. I hear you.

Bonobot

What concerns do you have about that?

SoHyun

I am afraid I may not be able to produce good research results.

Bonobot

That you feel afraid is understandable.

Bonobot

What worries about your current situation?

SoHyun

I don't have enough time for everything.

Bonobot

You don't have enough time for everything. Hmm.

Bonobot

Hmm. No need to worry if you don't.

Bonobot

Do you wish to be able to have enough time for everything?

SoHyun

Yes I wish I had 48 hours!

Bonobot

It is understandable that you wish to wish.

Bonobot

Many people wish as well.

Bonobot

Why do you want you had 48 hours?

SoHyun

Because I have so many things to do and 24 hours are not enough.

Bonobot

Possibly they are not enough.

Bonobot

Actually, I think you're already moving forward by talking about this.

Bonobot

Are you positive they are not enough?

SoHyun

In fact, I am not sure...

Bonobot

It's understandable you think you are not sure.

Bonobot

I think you being not sure is just like others.

Bonobot

What is your best hope now?

SoHyun

My hope is to be a competent researcher in my field.

Bonobot

I can tell. I've seen some stressed graduate students with similar problems.

Bonobot

Being a graduate student usually means a lot of stress and responsibilities.

Bonobot

What will it be like when the problem is solved?

SoHyun

I think will be with some quality published papers.

Bonobot

I see. you think will be with some quality published papers.

Bonobot

I think you're on the right track.

Bonobot

At this point, after reviewing all of this, what's the next step for you?

SoHyun

I think I have to focus on writing papers, instead of juggling with other chores.

Bonobot

Really?

SoHyun

I think so

Bonobot

We encounter all kinds of issues in life.

Bonobot

You know, they say action is the foundational key to success. So keep it up!

SoHyun

Thanks!

Bonobot

Oops, but the time's almost up. We've got to wrap up our conversation today.

SoHyun

Oh really?

Bonobot

So we talked about "your graduate school life" today. How do you feel after talking about it now?

SoHyun

Just a little bit better. I think I realized what my problem was.

Bonobot

Good to know. Thank you for coming in today. Let's talk again soon!

Bonobot

Bonobot has left the chat.
